# Inhibition of HIV-1 replication in primary human monocytes by the IκB-αS32/36A repressor of NF-κB

**DOI:** 10.1186/1742-4690-1-45

**Published:** 2004-12-21

**Authors:** Camillo Palmieri, Francesca Trimboli, Antimina Puca, Giuseppe Fiume, Giuseppe Scala, Ileana Quinto

**Affiliations:** 1 Department of Clinical and Experimental Medicine, University of Catanzaro "Magna Graecia", Via T. Campanella 115, 88100 Catanzaro, Italy; 2 Department of Biochemistry and Medical Biotechnology, University of Naples "Federico II", Via S. Pansini 5, 80131 Naples, Italy

## Abstract

**Background:**

The identification of the molecular mechanisms of human immunodeficiency virus type 1, HIV-1, transcriptional regulation is required to develop novel inhibitors of viral replication. NF-κB transacting factors strongly enhance the HIV/SIV expression in both epithelial and lymphoid cells. Controversial results have been reported on the requirement of NF-κB factors in distinct cell reservoirs, such as CD4-positive T lymphocytes and monocytes. We have previously shown that IκB-αS32/36A, a proteolysis-resistant inhibitor of NF-κB, potently inhibits the growth of HIV-1 and SIVmac239 in cell cultures and in the SIV macaque model of AIDS. To further extend these observations, we have generated NL(AD8)IκB-αS32/36A, a macrophage-tropic HIV-1 recombinant strain endowed to express IκB-αS32/36A.

**Results:**

In this work, we show that infection with NL(AD8)IκB-αS32/36A down-regulated the NF-κB DNA binding activity in cells. NL(AD8)IκB-αS32/36A was also highly attenuated for replication in cultures of human primary monocytes.

**Conclusions:**

These results point to a major requirement of NF-κB activation for the optimal replication of HIV-1 in monocytes and suggest that agents which interfere with NF-κB activity could counteract HIV-1 infection of monocytes-macrophages *in vivo*.

## Background

HIV-1 infection is characterized by a long period of clinical latency followed by the development of acquired immunodeficiency syndrome, AIDS. During latency and when viral replication is being controlled in patients treated with antiretroviral therapy, HIV-1 is present in cellular reservoirs and continues to replicate, with each ensuing round of replication giving rise to escape mutants, which further replenish viral reservoirs [1,2]. This grim picture calls for novel targeted therapies for eradicating virus-infected cells and for preventing new infections.

Initial infection *in vivo *by HIV-1 is thought to occur in CD4-positive, CCR5-positive lymphocytes and monocytes. Accordingly, when HIV-1 envelope protein in its oligomerized g160 form contacts the cell surface receptor a signalling cascade is triggered that results in transcriptional activation of specific gene arrays, such as the inflammatory cytokines IL-1 β, IL-6, IL-8, TNF-α, TGF-β; these cytokines, in turn, function to enhance the transcriptional activity of the proviral long terminal repeat (LTR) promoter [3,4]. This cytokine-driven inflammatory-like setting is mediated molecularly by the NF-κB family of transcription factors [5,6]; thus, it serves to reason that preventing NF-κB activation would attenuate HIV-1 replication. Indeed, the LTR of HIV-1 does contain two tandem NF-κB sites [7] and three repeated Sp1 sites [8] upstream of the TATAA box with an additional NF-κB site located in the 5' untranslated region of viral genome [9]. Both sets of NF-κB sequences enhance HIV-1 transcription in response to various signals [9]. However, the Sp1 sites and TATAA box can redundantly sustain the Tat-mediated transactivation of the HIV-1 LTR in the absence of NF-κB sites [10]. It is controversial whether NF-κB cellular factors are required for the HIV-1 replication. Mutant HIV-1 carrying deletions or base-pair substitutions in the NF-κB enhancer in the LTR have been shown to be either competent or incompetent for replication [11-13]. These divergent observations are likely explained by differing cellular contexts, such as primary cells versus immortalized cell lines, and varying levels of cellular activation.

IκB inhibitors regulate NF-κB activity [14]. In response to activating stimuli, IκB proteins become phosphorylated, ubiquinated and degraded by proteasomes. This releases cytoplasmic-sequestered NF-κB to enter the nucleus to activate the transcription of responsive genes [14]. The mutant IκB-αS32/36A is defective for serine 32- and serine 36-phosphorylation and is resistant to proteolysis. IκB-αS32/36A acts as a potent inhibitor of the NF-κB-dependent gene transcription, including those from the HIV-1 genome [15]. To verify the requirement of NF-κB in the replication of HIV-1 in primary cells, we previously designed HIV-1 and SIV molecular clones containing the IκB-αS32/36A cDNA positioned into the *nef *region of the respective viral genome [16,17]. We found that these recombinant viruses were highly attenuated for replication in T cell lines as well as in human and simian PHA-activated peripheral blood mononuclear cells, PBMCs [16,17]. These findings supported an interpretation that in these cellular contexts NF-κB is required for efficient viral replication. We also showed that a recombinant SIV which expressed IκB-αS32/36A inhibitor was also highly replication attenuated *in vivo *in rhesus macaque [17]. Here, we have extended our analysis of IκB-αS32/36A function in HIV-1 replication to primary monocytes. We report that a macrophage-tropic derivative of NL4-3 strain that expresses the proteolysis-resistant IκB-αS32/36A inhibitor of NF-κB replicated poorly in cultured primary human monocytes.

## Results

### Construction of pNL(AD8)IκB-αS32/36A

**Figure 1:**
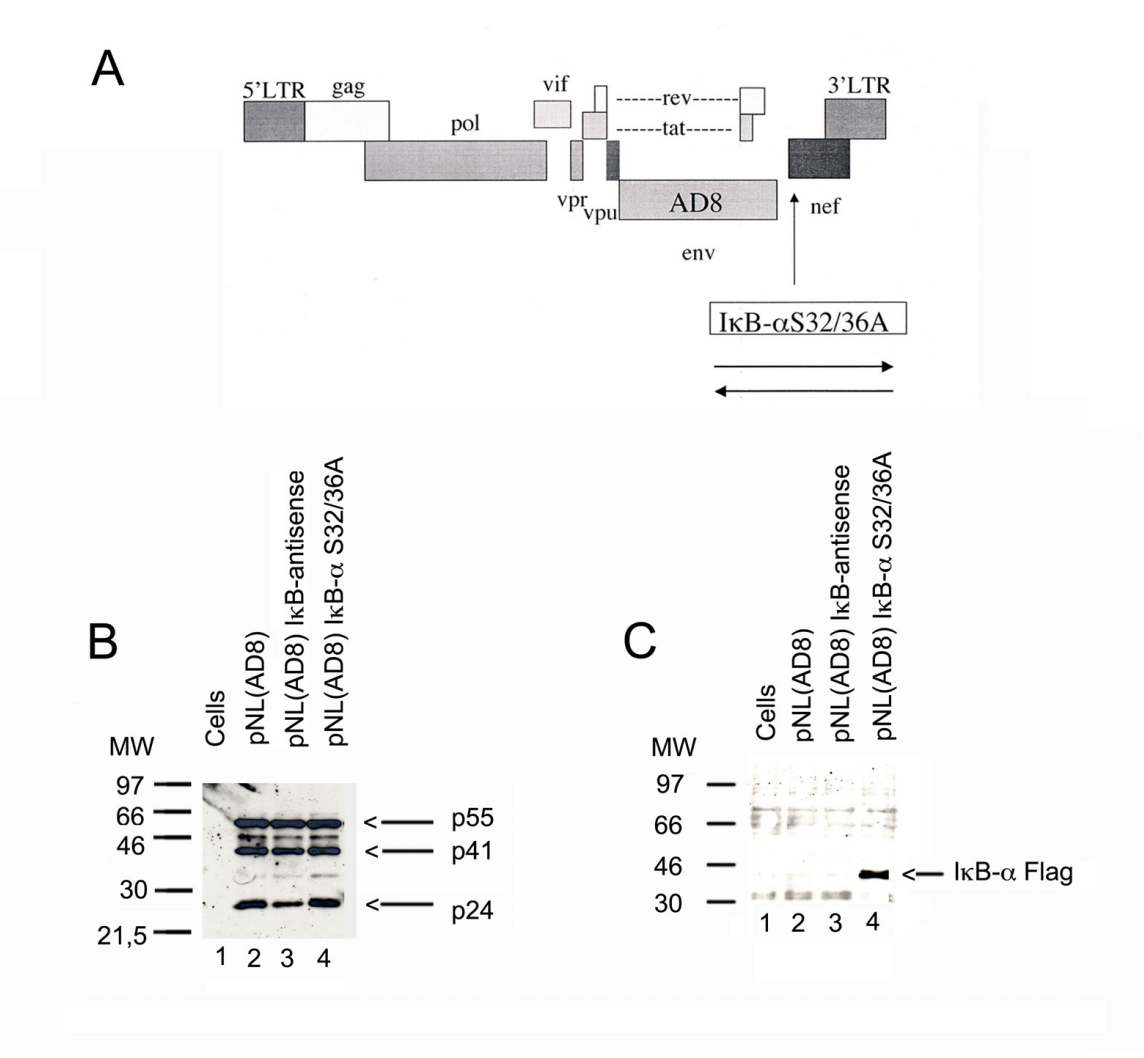
**Genome structure and expression of recombinant pNL(AD8)IκB-αS32/36A and pNL(AD8)IκB-antisense molecular genomes. **Panel A shows the structure of pNL(AD8) derivatives that carry the IκB-αS32/36A-FLAG insert into the *nef *region in sense (pNL(AD8)IκB-αS32/36A) or antisense (pNL(AD8)IκB-antisense) orientations. Panel B shows the immunoblot analysis using hyperimmune AIDS patient serum of total extracts (10 μg) from 293T cells 24 hours after transfection with the indicated viral plasmids (10 μg). Panel C shows the immunoblot analysis using an anti-FLAG monoclonal antibody of total extracts (10 μg) from 293T cells 24 h after transfection with the indicated viral plasmids (10 μg).

To generate a macrophage-tropic HIV-1 expressing the IκB-αS32/36A cDNA fused to the FLAG epitope, the CXCR4-tropic envelope of pNLIκB-αS32/36A [16] was replaced with the CCR5-tropic envelope from pNL(AD8) [18]. Briefly, the 2.7 Kb EcoR1-BamH1 fragment of pNL(AD8) was religated to the 13.1 Kb EcoR1-BamH1 fragment of pNLIκB-αS32/36A or pNLIκB-antisense, thus generating pNL(AD8)IκB-αS32/36A and pNL(AD8)IκB-antisense, respectively (Fig. 1A). Both molecular clones are Nef-minus because our cloning strategy deleted the first 39 amino acids from the N terminus of Nef and engineered a translational frameshift into the remaining Nef-encoding codons [16]. The respective molecular clones were transfected into 293T cells to analyse for the expression of HIV-1 proteins and IκB-αS32/36A polypeptide by immunoblotting (Fig. 1 B, C). As expected the IκB-αS32/36A-FLAG protein was expressed by pNL(AD8)IκB-αS32/36A (Fig. 1C, lane 4).

### Inhibition of NF-κB activity by pNL(AD8)IκB-αS32/36A

**Figure 2:**
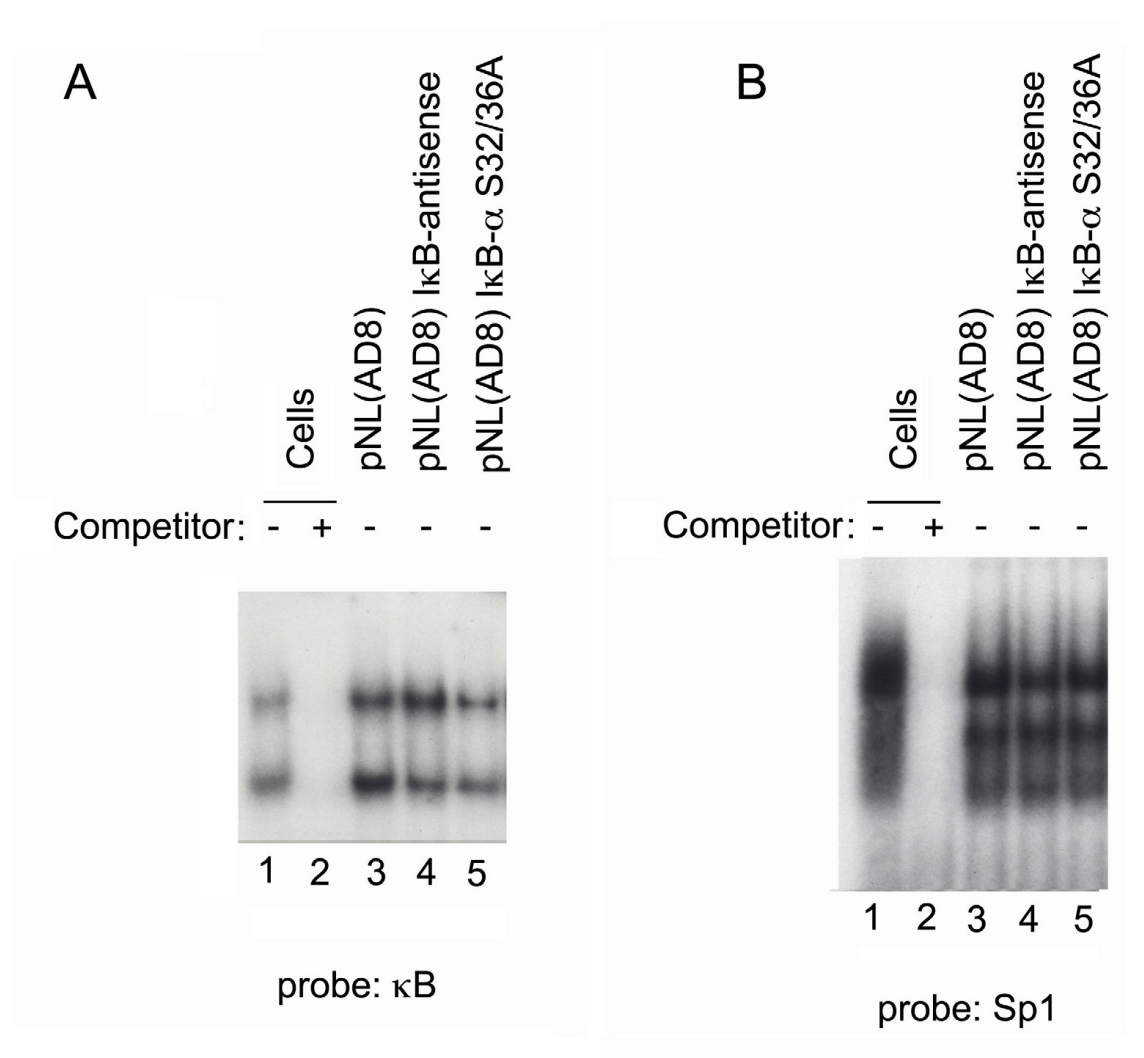
**Reduced NF-κB DNA binding activity in cells transfected with pNL(AD8)IκB-αS32/36A. **Panel A shows the NF-κB binding activity of nuclear extracts (5 μg) from 293 T cells transfected with the indicated viral plasmids (10 μg) or were mock-transfected. Panel C shows the Sp1 binding activity of the same nuclear extracts as in panel A. Binding competitions were performed with 100-fold molar excess of the respective unlabelled oligonucleotide.

To assess the functional impact of IκB-αS32/36A expressed from the recombinant NL(AD8) genome, 293T cells were transfected individually with pNL(AD8), pNL(AD8)IκB-αS32/36A or pNL(AD8)IκB-antisense, and the respective nuclear extracts were evaluated for NF-κB (Fig. 2A) and Sp1 DNA binding activity (Fig. 2B). A significant reduction in NF-κB DNA binding activity was observed upon transfection of pNL(AD8)IκB-αS32/36A (Fig. 2A, lane 5) as compared to the other viral transfections (Fig. 2A, lanes 3,4). The specificity of the IκBαS32/36A-mediated inhibition of NF-κB was verified by the demonstration that Sp1 binding to DNA was unaffected (Fig. 2B). These results support the interpretation that IκBαS32/36A expressed from the recombinant viral genome functionally inhibited NF-κB activity.

### Attenuation of pNL(AD8)IκB-αS32/36A in primary monocytes

**Figure 3:**
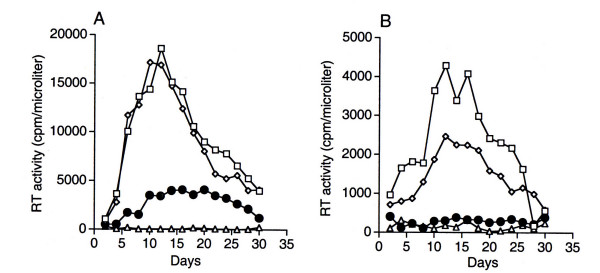
**Attenuated replication of NL(AD8)IκB-αS32/36A in primary human monocytes. **Panels A and B show the growth NL(AD8), NL(AD8)IκB-antisense and NL(AD8)IκB-αS32/36A in cultures of primary human monocytes. Cells (10^5^) were infected with equal amounts of viruses normalized based on RT counts of 10^6 ^cpm (A) or 10^5 ^cpm (B). A representative experiment of three independent infections of monocytes from different individuals is shown.

**Figure 4:**
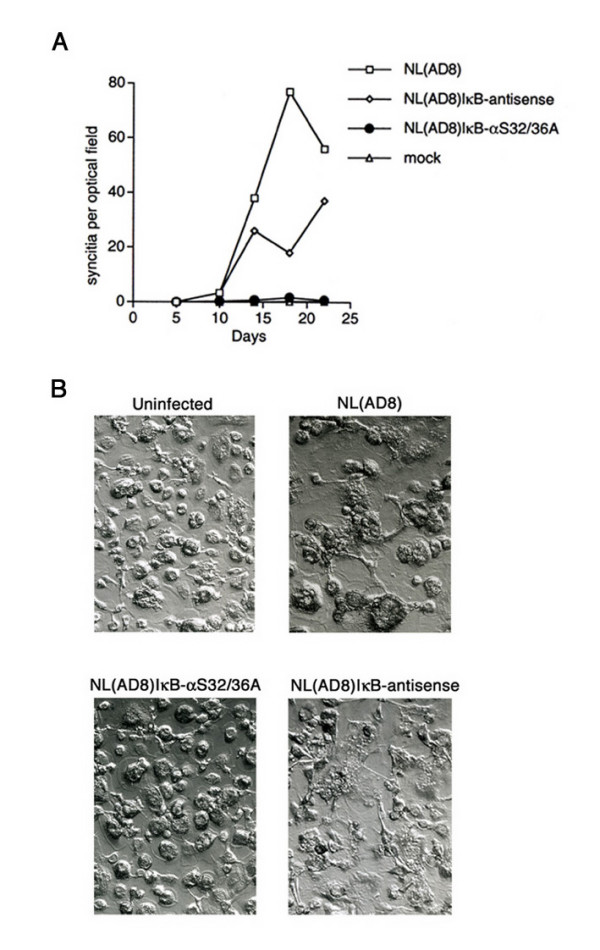
**Reduced syncitia formation by NL(AD8)IκB-αS32/36A in infection of primary human monocytes. **Panel A shows the kinetics of syncitia generation upon infection of primary human monocytes with 10^5 ^cpm RT activity of the indicated viral stocks. The average of syncitia observed per optical field is reported. Panel B shows the picture of primary human monocytes at 14 days post-infection with 10^5 ^cpm RT activity of the indicated viral stocks (original magnification × 430).

We next analyzed the replication properties of the recombinant HIV-1 genomes in cultured human monocytes from different individuals. Based on normalized amounts of input virus, we found that NL(AD8)IκB-αS32/36A was highly attenuated for replication when compared to NL(AD8) and NL(AD8)IκB-antisense (Fig. 3 A-B). Accordingly, virus-induced syncitium formation was also strongly inhibited in monocytes infected with NL(AD8)IκB-aS32/36A (Fig. 4 A, B). Taken together, our results underscore a critical contribution of NF-κB to HIV-1 growth in monocytes.

## Discussion

Substantial numbers of monocytes are preserved in infected individuals even at later clinical stages of AIDS, when T cell numbers are dramatically reduced. Consistently, in animal models of HIV-1 infection, monocytes are the major reservoir after acute depletion of CD4-positive T cells [19,20]. This indicates that these cells are long lasting infected moieties that shuttle from mucosal sites to lymph nodes and could function as a major HIV-1 reservoir *in vivo*. In addition, monocytes are programmed to produce a large amount of inflammatory cytokine, including IL1-β, IL-6, TNF-α, which are strong inducers of HIV-1 replication [5]. Indeed, HIV-1 envelope binding to CCR5 receptor activates an intracellular signalling cascade that promotes high levels of transcription factors, including NF-κB, which sustain the initial rounds of viral replication and induce the production of inflammatory cytokines which activate surrounding cells to become more susceptible to virus infection [3,4].

Based on the published literature, the role of NF-κB in HIV-1 replication has been controversial [13,16,21]. For instance, the deletion of NF-κB binding sites from HIV-1 and SIV LTRs [22] has suggested that NF-κB activity may not be required for HIV-1 LTR-directed transcription. Moreover, deletion of NF-κB sequences in the LTR has also been reported not to affect HIV-1 replication in defined cellular settings [11,12]. These latter studies relied on short-term infections of immortalized cells that may not express a physiologic concentration of transcription factors. To address this issue, we have developed a novel HIV-1 strain, NL(AD8)IκB-αS32/36A, which was engineered to express a proteolysis-resistant IκBαS32/36A, and is a strong inhibitor of NF-κB activity. This recombinant virus expresses the envelope of the AD8 strain, a macrophage-tropic virus. Our findings show that NL(AD8)IκB-αS32/36A replication profile is different from that of the NL(AD8)IκB-antisense control. NL(AD8)IκB-αS32/36A failed to produce a productive infection in primary monocytic cells over a thirty-days acute infection (Fig. 3). These results were correlated with a strong inhibition NF-κB activity in NL(AD8)IκB-αS32/36A-infected cells (Fig. 2), indicating that in the setting of HIV infection of primary monocytes NF-κB plays a non-redundant role. These results are in agreement with the evidence that IκB-αS32/36A negatively affected the replication of HIV and SIV in PBMC cultures and in monkeys [16,17].

Because IκB-αS32/36A constitutively inhibits NF-κB [15], the potent inhibition of HIV/SIV replication could be due to repression of the NF-κB-dependent activation of HIV/SIV transcription. However, additional mechanisms might explain the potent inhibition of HIV/SIV replication by IκB-αS32/36A. In this regard, IκB-α regulates the transcriptional activity of NF-κB-independent genes by interacting with nuclear co-repressors, histone acetyltransferases and deacetylases [23,24]. Further studies are required to clarify novel activities of IκB-α in the modulation of the transcriptional machinery. Our results underscore a central role for IκB-α as a potent inhibitor of the replication of HIV-1 in both T cells [16] and monocytes (this study), and point to the NF-κB/IκB network as a suitable target for therapeutic intervention of AIDS.

## Conclusions

In this study we have addressed the role of NF-κB/IκB proteins in the replication of HIV-1 in primary human monocytes. We show a strong attenuation in the replication of a macrophage-tropic HIV-1 strain expressing the IκB-αS32/36A repressor of NF-κB in primary cultures of human monocytes. These results are consistent with previous evidence of HIV/SIV inhibition by IκB-αS32/36A in PBMCs and in macaques [16,17]. In addition, these findings further support a role of NF-κB inhibitors in blocking HIV-1 replication and suggest novel strategies for the development of anti-viral therapy that targets NF-κB factors.

## Methods

### Transfections and Viral stocks

293T cells were cultured in Dulbecco's modified Eagle's medium supplemented with 10% v/v heat-inactivated fetal bovine serum and 3 mM glutamine. Viral stocks were produced by transfecting 293T cells (10^6^) with viral plasmids (10 μg) using calcium phosphate. Forty hours later, the cell culture supernatant was passed through a 0.45-μm filter and measured for RT activity as previously described [16].

### Immunoblotting analysis

293T cells were transfected with viral plasmids (10 μg) and lysed in RIPA buffer (150 mM NaCl, 1 % Nonidet P-40, 0.5 % sodium deoxycholate, 0.1% sodium dodecyl sulfate, 50 mM Tris-HCl pH 8.0) 24 hours later. Proteins (10μg) were separated by electrophoresis in 10% SDS-polyacrylamide gel and transferred to Immobilon-P (Millipore). Filters were blotted with an AIDS patient serum or with anti-FLAG monoclonal antibody by using Western-Light Chemiluminescent Detection System (Tropix, Bedford, MA).

### Electrophoretic Mobility Shift Assays

Nuclear extracts and gel retardation assays were performed as described previously [9]. Briefly, cells were harvested, washed twice in cold phosphate-buffered saline, and resuspended in lysing buffer (10 mM Hepes, pH 7.9, 1 mM EDTA, 60 mM KCl, 1 mM DTT, 1 mM phenylmethylsulfonyl fluoride, 0.2% v/v Nonidet P-40) for 5 min. Nuclei were collected by centrifugation (500 × *g*, 5 min), rinsed with Nonidet P-40-free lysing buffer, and resuspended in 150 μl of buffer containing 250 mM Tris-HCl, pH 7.8, 20% glycerol, 60 mM KCl, 1 mM DTT, 1 mM phenylmethylsulfonyl fluoride. Nuclei were then subjected to three cycles of freezing and thawing. The suspension was cleared by centrifugation (7000 × *g*, 15 min), and aliquots were immediately tested in gel retardation assay or stored in liquid phase N2 until use. The HIV-1 NF-κB oligonucleotide probe was 5'-CAAGGGACTTTCCGCTGGGGACTTTCCAG-3'; the Sp1 oligonucleotide probe was 5'-GGGAGGTGTGGCCTGGGCGGGACTGGGGAGTGGCG-3'. The probes were end-labelled with [γ-^32^P]ATP (Amersham Int., Buckinghamshire, UK) using polynucleotide kinase (New England Biolabs, Beverly, MA). Equal amounts (5 μg) of cell extracts were incubated in a 20 μl reaction mixture containing 10% glycerol, 60 mM KCl, 1 mM EDTA, 1 mM DTT, and 2 μg of poly [d(G-C)] (Boehringer Mannheim, Germany) for 5 min on ice. One μl of [γ^32^P]-labelled double-stranded probe (0.2 ng, 5 × 10^4 ^cpm) was then added with or without a 100-fold molar excess of competitor oligonucleotide. The reactions were incubated at room temperature for 15 min and run on a 6% acrylamide:bisacrylamide (30:1) gel in 22.5 mM Tris borate, 0.5 mM EDTA. Gels were dried and autoradiographed.

### Monocytes cultures and infections

Human monocytes were isolated from PBMC by elutriation, cultured in RPMI, 10% FCS and GMCSF (20 ng/ml) for 48 hours. Infections were performed with viral stocks measured by reverse-transcriptase (RT) activity [16]. Usually, cell cultures (10^5 ^cells) were infected with 10^5 ^- 10^6 ^cpm of RT activity. The cell culture supernatants were collected every two days and replaced with fresh medium. The viral production was measured as RT activity in the culture supernatants as previously described [16]. The syncitia formation in cell cultures was evaluated by calculating the average number of syncitia in at least six optical fields.

## List of abbreviations used

NF-κB, nuclear factor kappa B

IκB, inhibitor of nuclear factor kappa B

IL-1, interleukin-1

IL-6, interleukin-6

IL-8, interleukin-8

TNF-α, tumor necrosis factor alpha

TGF-β, transforming growth factor-beta

cpm, counts per minute

FCS, fetal calf serum

GMCSF, granulocyte-macrophage colony-stimulating factor

## Competing interests

The author(s) declare that they have no competing interests.

## Authors' contributions

CP carried out the analysis of viral growth and DNA band-shift assays. FT was responsible for cell cultures. AP performed the immunoblotting analysis. GF produced the viral plasmids and viral stocks, and performed the artwork of the paper. GS participated in the design of the study and discussion of results. IQ designed this study and edited the manuscript.
